# Railroad Sleeper Condition Monitoring Using Non-Contact in Motion Ultrasonic Ranging and Machine Learning-Based Image Processing

**DOI:** 10.3390/s23063105

**Published:** 2023-03-14

**Authors:** Diptojit Datta, Ali Zare Hosseinzadeh, Ranting Cui, Francesco Lanza di Scalea

**Affiliations:** Experimental Mechanics, NDE & SHM Laboratory, Department of Structural Engineering, University of California San Diego, La Jolla, CA 92093, USA; d1datta@eng.ucsd.edu (D.D.); hosseinzadeh@eng.ucsd.edu (A.Z.H.);

**Keywords:** railroad sleeper monitoring, ultrasonic ranging, digital signal processing, machine learning and image processing, nondestructive evaluation

## Abstract

An ultrasonic sonar-based ranging technique is introduced for measuring full-field railroad crosstie (sleeper) deflections. Tie deflection measurements have numerous applications, such as detecting degrading ballast support conditions and evaluating sleeper or track stiffness. The proposed technique utilizes an array of air-coupled ultrasonic transducers oriented parallel to the tie, capable of “in-motion” contactless inspections. The transducers are used in pulse-echo mode, and the distance between the transducer and the tie surface is computed by tracking the time-of-flight of the reflected waveforms from the tie surface. An adaptive, reference-based cross-correlation operation is used to compute the relative tie deflections. Multiple measurements along the width of the tie allow the measurement of twisting deformations and longitudinal deflections (3D deflections). Computer vision-based image classification techniques are also utilized for demarcating tie boundaries and tracking the spatial location of measurements along the direction of train movement. Results from field tests, conducted at walking speed at a BNSF train yard in San Diego, CA, with a loaded train car are presented. The tie deflection accuracy and repeatability analyses indicate the potential of the technique to extract full-field tie deflections in a non-contact manner. Further developments are needed to enable measurements at higher speeds.

## 1. Introduction

Structural components undergo deterioration during their service life due to numerous factors such as fatigue from repeated loading and unloading, exposure to environmental conditions, aging, etc. Structural health monitoring (SHM) [1] of such aging structures is essential to prevent catastrophic failures and ensure their safety and reliability. Non-destructive evaluation (NDE) and SHM techniques are widely being used to ensure safety and reliability during the service life of various structural components in the aerospace [2,3,4], civil [5,6,7,8], and railroad industries [9,10,11]. One of the key challenges in the SHM community is the lack of rapid inspection techniques that facilitate the frequent and autonomous inspection of structural components.

Rapid inspection techniques are of particular interest to the railroad industry which transports more than 10,000 billion freight ton-kilometers and 3000 billion passenger-kilometers every year around the world [12]. Railroad tracks are complex systems consisting of rails, railroad ties (sleepers), fasteners, the ballast, and an underlying subgrade. The primary functions of railroad ties are (1) to support the rails to maintain gauge distance and vertical rail alignment, (2) to transfer loads uniformly from the rails to the underlying ballast, and (3) to provide restraint from longitudinal and lateral rail movements [13]. The ballast helps to provide load distribution over a larger area from ties to the underlying subgrade, energy absorption from static and dynamic train loads, and water drainage. The ballast consists of large and consistently sized aggregates that are uniformly in contact with the bottom tie surface to ensure seamless load distribution. However, in certain conditions, when only a small number of ballast grains are in contact with the tie surface, high contact stresses are developed [14]. These localized stress concentrations, especially near the tie ends, can lead to tie-bottom abrasion, ballast pulverization, and voids in the tie-ballast interface. Under these circumstances, the ballast tends to lose contact near the ends of the tie and binds to the center—a condition known as “center-binding support condition”. Such a support condition can lead to premature tie failures, resulting in train derailments. Common failure modes for railroad concrete ties [15,16,17,18,19,20,21,22,23] include central negative flexural cracking and abrasion along the tie–ballast interface. It has been shown that center binding support conditions can be detected from the deflection shapes of ties under loads [24,25]. Ties with center binding conditions tend to develop negative bending (tension on the top surface) deflections under train loads. Such deflections produce flexural cracks on the tie surface, leading to increased corrosion and eventually broken ties. Tie deflections can also be used for measuring vertical relative rail seat displacement, which is an important metric to indicate the bending failure of concrete and wood ties.

Several techniques have been proposed by researchers for measuring vertical tie deflections [26,27,28,29,30,31]. Frohling [32] proposed using a linear variable differential transformer (LVDT). The drawback of LVDTs is that they require a fixed reference point with respect to which the deflections can be measured. Finding a fixed reference point can be challenging near a track because of relative displacements produced by train loads in the surrounding locations [33]. Kim et al. [34] introduced a technique based on firing a laser beam at a photosensitive receptor placed on the tie surface. Priest et al. [35] mounted geophones on the ends of a railroad tie to measure vertical velocities under train loads. Tie deflections were then obtained with a single integration step on the acquired velocity data. Mounting accelerometers on the tie and using a double integration technique to compute tie deflections has been suggested by several researchers [26,27,28,29,30,31,32,33]. Accelerometer-based deflection measurements, however, are mainly sensitive to high frequency deflections. Therefore, Joh et al. [36] suggested using strain gages (for low frequency deflections) along with accelerometers (for high frequency deflections) mounted on ties. These techniques are not full-field and only provide deflection data points where transducers are located. Moreover, they either rely on contact-based sensing techniques or need a clean surface for accurate operation. As a result, such techniques cannot be used for automatic in-motion tie tracking purposes. Digital image correlation (DIC)-based techniques have been proposed as an alternative to transducer mounted techniques, which could facilitate non-contact and in-motion inspections [37,38]. Such techniques require careful calibration between the pixel and physical domains to ensure accurate measurements. Results for DIC-based techniques are also susceptible to variations from lighting conditions, lens aberrations and distortions.

This study introduces a railroad tie monitoring system using an ultrasonic sonar-based ranging technique. A transducer array, placed parallel to the railroad tie, is used in pulse-echo mode and full-field tie deflections profiles are reconstructed in 3D using a reference-based cross-correlation approach. In-motion field tests were performed at walking speed by mounting an inspection prototype on a loaded train car at a BNSF yard in San Diego, CA, USA.

The paper is structured as follows. Section 2 provides a background of the ultrasonic technique for evaluating tie deflections. Section 3 describes the signal processing routines used for reference-based tie deflection measurements. The data acquisition system and field test setup are discussed in Section 4, followed by Section 5, where a machine learning-based image processing technique is leveraged to demarcate tie boundaries and to track spatial locations of the tie tracking system along the train movement direction. Section 6 presents the results from the field tests conducted at a BNSF yard by mounting a prototype on a loaded train car. Lastly, the concluding remarks and plans for future tests are outlined in Section 7.

## 2. Background and Main Contribution

**Figure 1:**
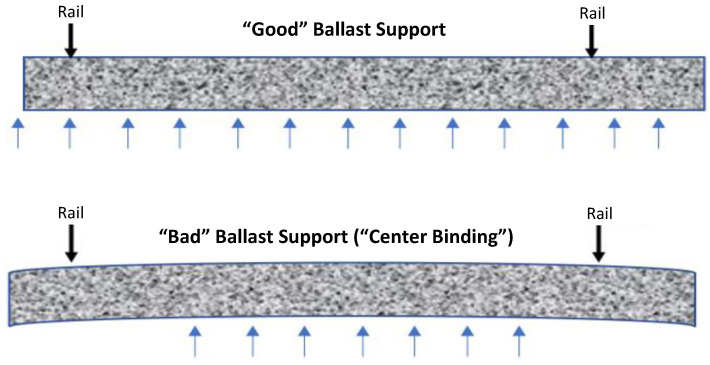
Deflected shape profiles for good and bad ballast support conditions.

Railroad ties with center-binding ballast support conditions produce a negative flexural bending as shown in Figure 1, which can potentially be identified by tracking the deflection profile of the tie under service loads.

**Figure 2:**
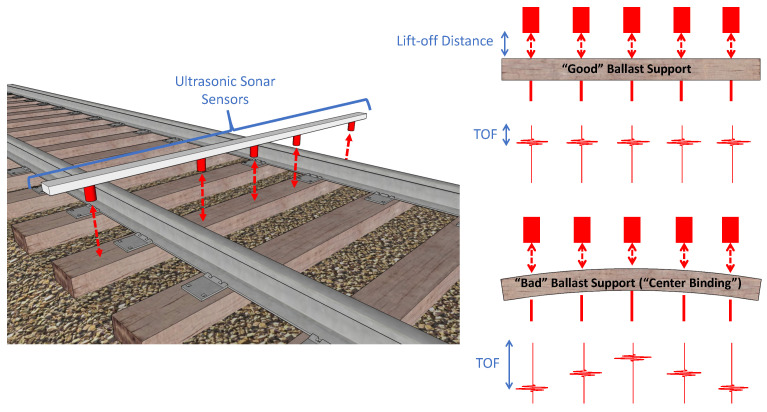
Concept of ultrasonic ranging for measuring the vertical deflection of a tie using time-of-flight (TOF).

This study utilizes a non-contact acoustic technique in “sonar” mode to track the spatial position of selected points at the top surface of a tie to calculate the tie’s deflection *in motion*. The proposed technique utilizes known concepts of acoustic ranging, including time-of-flight (TOF) tracking and signal detection, and adapts them to the specific application of tie deflection measurements. An array of non-contact acoustic transducers is mounted on a beam (schematically shown in Figure 2) that is rigidly connected to the frame of a train car.

**Figure 3:**
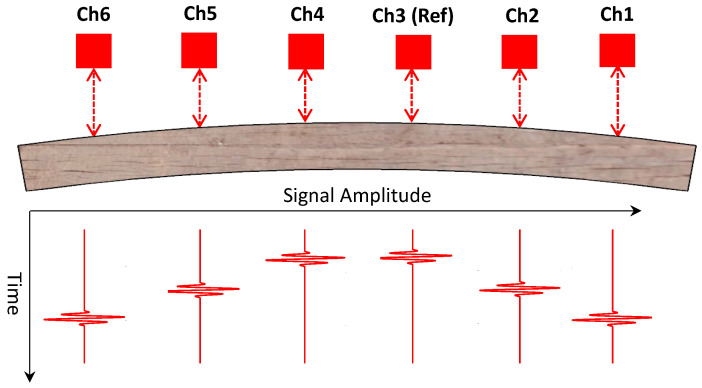
Schematic diagram of transducer array and corresponding waveforms for a tie with center-binding (negative bending).

The transducers continuously excite an acoustic signal directed toward the ties. The acoustic signals reflect from the top surface of the tie and are measured by the same transducers (i.e., pulse–echo operation) as shown in Figure 3.

**Figure 4:**
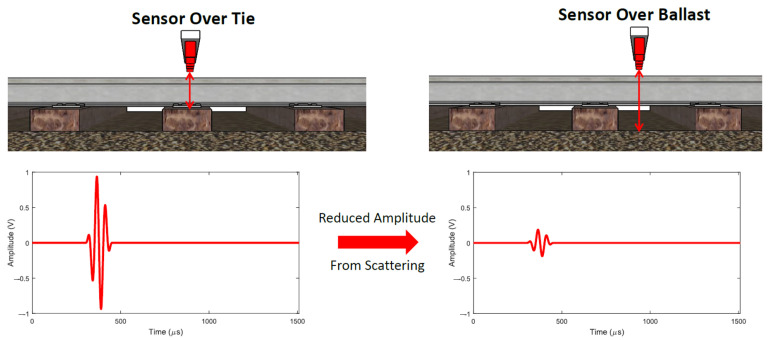
Difference in reflected waveform amplitude from the tie and ballast.

Since the speed of sound in air is known (v= 343 m/s), the distance of the tie from a typical transducer (i.e., lift-off distance), *d*, can be easily calculated as:

(1) $$d=\frac{t\times v}{2}$$

where *t* is the TOF of the reflected wave from the tie surface. The factor of 2 considers the distance travelled by the wave from the transducer to the tie surface and back to the transducer. However, this measurement does not directly give the tie displacement at that transducer location. This computed lift-off distance varies based on the difference in tie surface elevations of successive ties. A tie sitting lower on the ballast than the adjacent tie would result in a higher computed lift-off distance since the transducers are held at the same elevation. To overcome these variabilities, one of the transducer channels is assigned as a reference. The difference in TOFs with respect to this reference transducer is computed for all other transducer locations and the distance corresponding to this difference in TOF gives the actual deflection at that location with respect to the reference surface. One way to compute this reference-based deflection would be to compute the lift-off distances at each channel location through peak-detection techniques based on signal gating and thresholding, and subtracting the reference transducer lift-off distance from the other transducer lift-off distances. The problem with such a technique is that it relies on peak detection with thresholding and the received pulse has multiple peaks where the amplitudes depend on the tie surface (rough surfaces result in lower amplitude signals) and the lift-off distance (higher lift-off distances result in lower amplitudes due to attenuation). Wave dispersion could also affect the peak detection since air is a mildly dispersive medium (due to frequency-dependent attenuation) and the signal pulse tends to spread out in time as it travels through space. To overcome these challenges, a cross-correlation-based difference TOF computation technique [39,40] was used instead, as discussed in Section 3. The cross-correlation operator matches waveform shapes instead of tracking individual peaks to compute the TOF and is therefore more accurate. The transducer array is used in motion to scan over the ties through multiple pulse–echo measurements. Multiple measurements along the tie width enable the measurement of twisting deformations along with the longitudinal deformations. Note that based on the speed of the acoustic waves in air and an average lift-off distance of 11 inches, the measurement sampling rate along the direction of in-motion scans is limited to a repetition rate of 600 Hz, returning a resolution of approximately a quarter inch along the tie width. In-motion scanning of ties requires discarding waveform reflections from the ballast to demarcate tie boundaries. The amplitudes of the reflected waveforms from the ties and ballast differ considerably due to differences in morphology. The ballast acts like a wave scatterer and the reflected signals are much weaker compared to the reflections from tie surfaces (shown in Figure 4).

Therefore, an acoustic waveform amplitude-based classifier can be used to discard waveforms from the ballast and demarcate tie boundaries. Despite the overall good performance of such an object classification technique, it is possible that ballast areas are misclassified as ties. To improve the classification, a high frame-rate camera was added to the sensing layout to grab images of the probed objects, followed by machine learning-based object classification. The images can be also further analyzed to estimate the spatial location of the probed objects, which is needed to reconstruct the deflection profile of the ties, especially if the tests are performed without using traditional positioning systems (e.g., tachometers).

## 3. Cross-Correlation-Based Time-of-Flight Measurement

**Figure 5:**
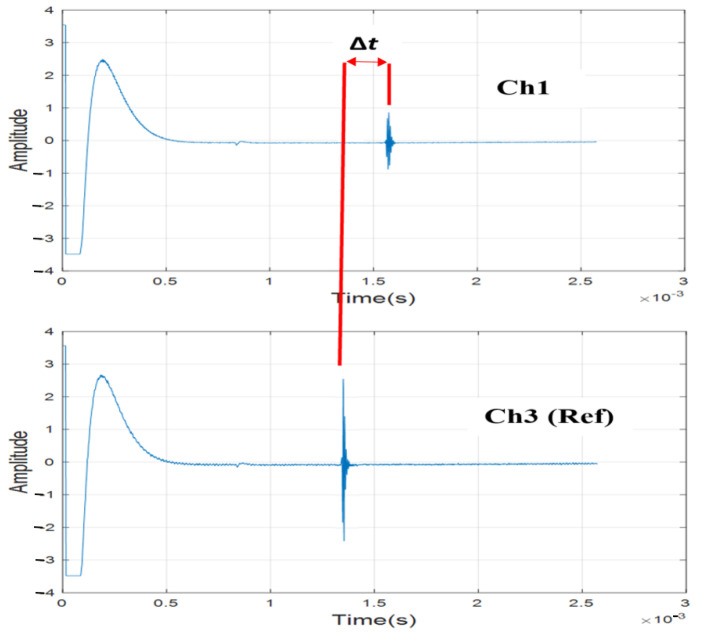
Recorded waveforms at two transducer locations (1 and 3) showing the difference in TOFs.

Consider a tie with a negative flexural bending as shown in Figure 3. The transducer array consists of six ultrasonic air-coupled transducers in pulse-echo mode. The recorded waveforms from transducer 1 (*Ch*1) and transducer 3 (*Ch*3) are shown in Figure 5.

**Figure 6:**
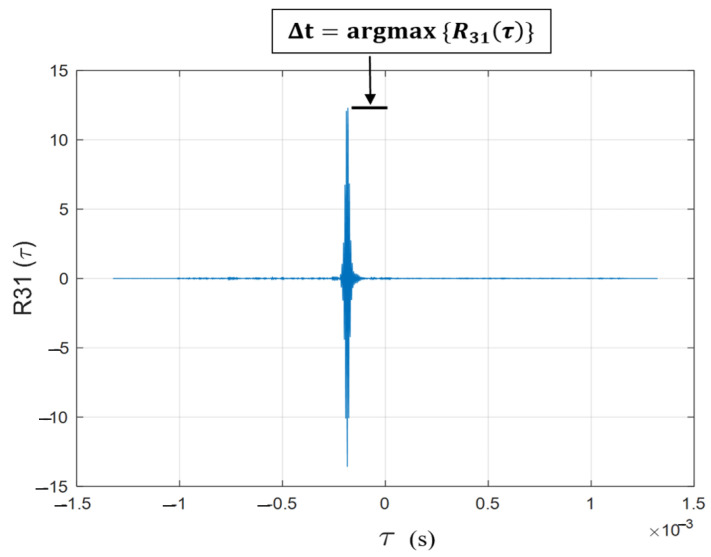
Cross-correlation function between waveforms from two locations on the tie (1 and 3) to compute the differential TOF.

Transducer 3, located near the center, is used as the reference, with respect to which the deflections at all other locations on the tie will be measured. Note that the TOF for *Ch*1 is higher than *Ch*3 (see Figure 5) since *Ch*1 is farther away from the tie surface than *Ch*3 for the deflection shape shown in Figure 3. The aim is to find the difference in the TOF (denoted by Δt in Figure 5). Δt is proportional to the deflection at *Ch*1 with respect to *Ch*3. To compute Δt, a cross-correlation operation is used. Consider $x_{1}\left(t\right)$ be the time-series signal at transducer 1, and $x_{3}\left(t\right)$ to be the time-series signal at transducer 3 (reference). The cross-correlation operator $R_{31}\left(\tau \right)$ is defined as:

(2) $$R_{31}\left(\tau \right)=\sum_{t=-\infty }^{+\infty }x_{3}\left(t\right)x_{1}\left(t-\tau \right)$$

where τ is the variable time-delay parameter used in the cross-correlation function. Since both $x_{1}\left(t\right)$ and $x_{3}\left(t\right)$ are finite time signals of say length (*T*), the expression in Equation (2) can be simplified as:

(3) $$R_{31}\left(\tau \right)=\sum_{t=0}^{T}x_{3}\left(t\right)x_{1}\left(t-\tau \right)$$

and Δt can then be calculated as:

(4) $$\Delta t=argmax\{R_{31}\left(\tau \right)\}$$

where ‘argmax’ is the *argument of the maxima operator* and returns the τ at which $R_{31}\left(\tau \right)$ reaches its maximum value. Deflection at *Ch*1 with respect to *Ch*3 is then computed as follows:

(5) $$\Delta d_{1}=\frac{\Delta t\times v}{2}$$

where *v* is the speed of sound in air. Figure 6 shows the cross-correlation series for $R_{31}\left(\tau \right)$ computed for the signals in Figure 5.

This cross-correlation is performed for all transducer locations with respect to the same reference (i.e., *Ch*3) to compute $\Delta d_{1}$, $\Delta d_{2}$, $\Delta d_{3}$, $\Delta d_{4}$, $\Delta d_{5}$ and $\Delta d_{6}$. Note that $\Delta d_{3}$ is the deflection at the reference channel (*Ch*3) and it is equal to zero. The cross-correlation is performed by gating both the signals between 1.25 ms and 2.25 ms (T=1 ms), which is a time window within which the reflected signal is expected based on the average distance between the tie and the transducer array (11 inches). The gated signal is passed through a Butterworth high-pass filter with a cut-off frequency of 10 kHz to eliminate the low-frequency noises induced by the train vibrations. Referring to Figure 6, note that Δt is negative, indicating that the surface elevation of the tie at transducer location 1 (*Ch*1) is lower than the elevation at transducer location 3 (*Ch*3). It should also be mentioned that the amplitude of the waveform at *Ch*1 is smaller than at *Ch*3, as shown in Figure 5. This is because the deflection shape of the tie produces different angles of the incident wave at these two locations. The incident wave angle at *Ch*3 is more normal to the tie and produces a stronger reflection than the wave at *Ch*1.

## 4. Data Acquisition System and Field Test Setup

**Figure 7:**
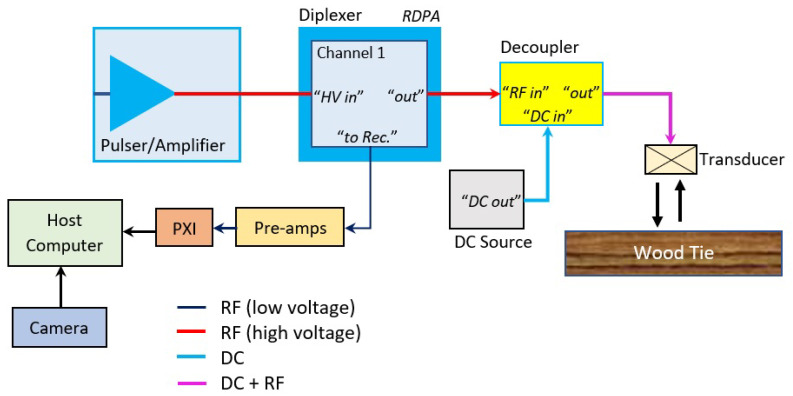
Schematic of data acquisition system (1 channel setup) with a camera.

A schematic diagram of the data acquisition system (one channel) using capacitive air-coupled ultrasonic transducers is shown in Figure 7.

Six transducers (VN Instruments CAP-2) with a central frequency of 120 kHz were used, along with a high frame rate camera (Basler, acA1300-200uc (USB-3.0)). The setup consists of a pulser/amplifier unit which sends out excitation pulses (∼100 V) to the transducers, which triggers it to transmit the outgoing pulse to the tie surface. The excitation pulse from the pulsing unit also triggers the data acquisition system programmed in National Instruments (NI) LabVIEW Real-Time. The pulser unit was programmed to trigger the data acquisition system at a repetition rate of 100 Hz. The high energy excitation pulse and the low energy received signal are on the same channel, and the diplexer (Ritec RDX-SD-2X) splits these two signals and prevents the high energy excitation signal from leaking into the receiving channel. The capacitive transducers require a DC bias of 200 V during operation. The AC/DC decoupler combines the excitation signal and the DC bias on the outgoing channel to the transducers and also prevents the DC bias from leaking into the receiving channel to the diplexer. The received signal from the diplexers is fed to pre-amplifiers (Olympus 5662), which amplify the signals before they are sent to the NI PXI 5105 data acquisition card. The data acquisition system is programmed in LabVIEW Real-Time running on a NI PXIe-8135 processor. The real-time processor analyzes the received signals in parallel with the acquisition. A host computer is used to run the LabVIEW code in the target PXI device. A small holder is used to rigidly attach the camera to the prototype beam, such that its field of view is focused on the probed objects by the sensing heads. A USB-3.0 connection is established to connect the camera to the host computer via a LabVIEW module. A software-level trigger is used to continuously activate and grab images as the train car moves, while images are saved based on single input-single output (SISO) network-wise communication, originated by the signal acquisition LabVIEW module. Therefore, a synchronized set of images and waveforms is saved for future analysis. Although this configuration is optimal for the present application, some of the images were missed in the saving loop due to the inherent buffer limitations of the USB-3.0-based data transmission protocol.

**Figure 8:**
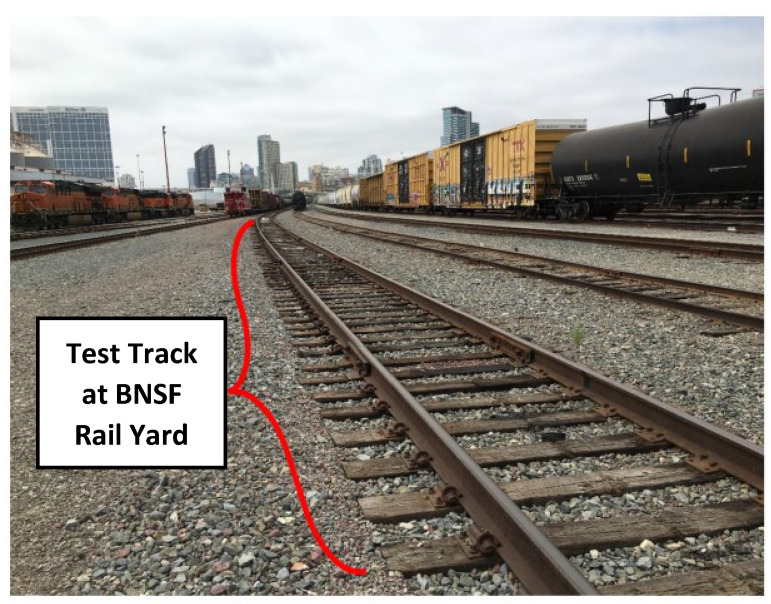
Section of test track at a BNSF rail yard in San Diego, CA, USA.

**Figure 9:**
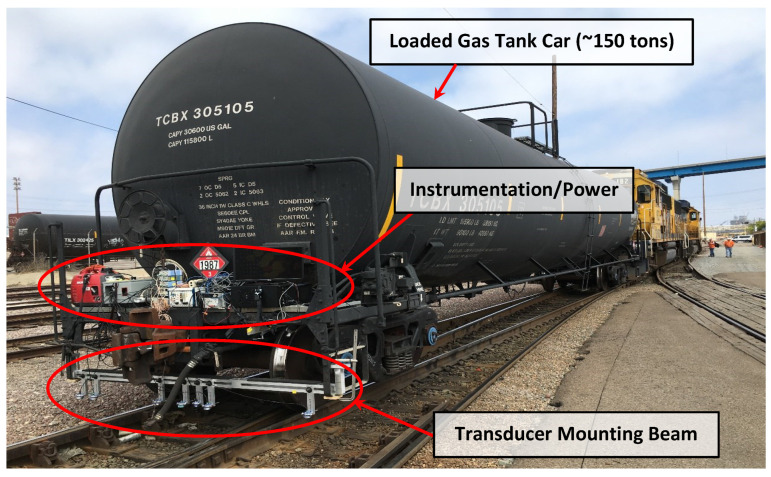
Test setup for in-motion tie deflection measurements at the BNSF rail yard in San Diego, CA, USA.

Tests were performed on a test track (see Figure 8) at a BNSF rail yard in San Diego, CA, USA, by mounting a prototype on a loaded gas tank car, as shown in Figure 9.

Test runs were performed at walking speed (∼5 mph) over a tangent section of track with wood ties. A total of 150 ties were scanned during each test run. Data acquisition was stopped after the last tie was scanned, and the train was brought back to the first tie to begin the next test run. A total of four test runs were performed and the same ties were scanned during each test run.

**Figure 10:**
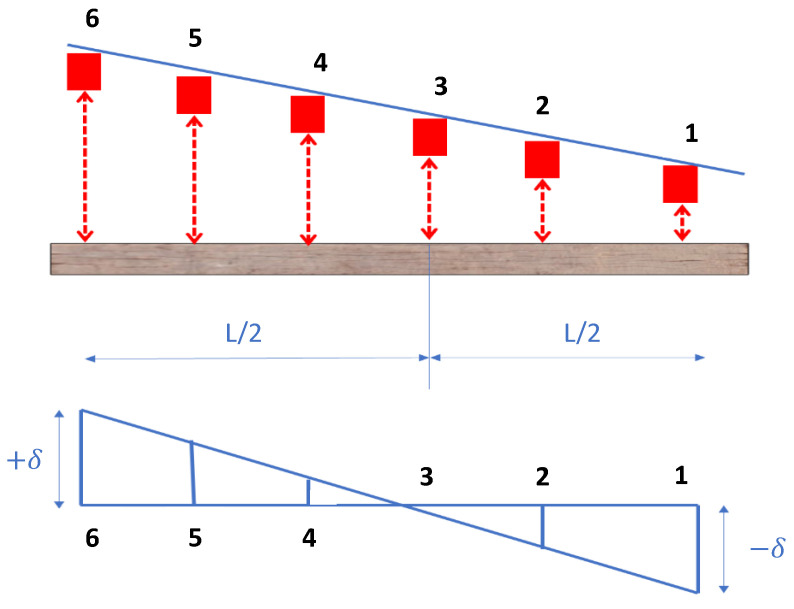
Bias removal from tie deflection measurements.

One concern that was observed during the tests was a misalignment of the transducer beam that introduced some bias in the deflection measurements. This type of misalignment occurred because the transducer on one end of the beam was sitting lower than the transducer at the other end of the beam. Figure 10 shows a schematic of the issue.

Based on this figure, transducer 1 on one end of the beam sits lower than transducer 6 on the other end because the transducer mounting beam is not perfectly horizontal. Such an arrangement produces spurious deflections even if the tie is perfectly straight, as depicted in Figure 10, where transducer 1 measures the deflection in the downward direction (−δ) and transducer 6 measures the deflection in the upward direction (+δ). Similarly, the deflections at transducer locations 2, 4 and 5 are also off and a linear variation in spurious deflections is observed. The magnitude of bias (δ) at transducer locations 1 and 6 is the same because they are equidistant from the center of the mounting beam, where the reference transducer is located (location 3). To remove the bias, the average difference in deflections at transducer locations 1 and 6 was computed over several different ties and this computed value in the average sense would converge to 2δ (assuming the two ends of the tie would be at the same level in an averaged sense over a number of ties). Once the magnitude of the bias is determined at the two ends of the beam (δ), a bias negating value is added to each transducer deflection measurement to effectively remove the bias. For example, the bias negative value at locations 1 and 6 would be +δ and −δ, respectively. The bias negating values at locations 2, 4 and 5 can be determined from similarity of triangles, since the distances between transducer locations are known. All results presented in this paper include the bias removal technique discussed here. In future tests, this bias could be eliminated by calibrating the horizontality of the transducer mounting beam. Although, note that the presence of this bias would not affect the shape of the deformed tie and would rather appear as a rigid body rotation about an axis parallel to the railroad track.

## 5. Image-Based Techniques for Spatial Position Tracking and Object Classification

By processing the captured images, two main goals are achieved. First, the travel distance of the train is extracted by tracking robust features in the successive images, which are subsequently used to determine the spatial position of the proposed sonar system. Note that image-based spatial positioning was used in lieu of an encoder. Second, a machine learning-based object classification is performed to supplement the acoustic signal-based tie boundary identification. In the following, the different steps of the image processing techniques are described.

**Figure 11:**
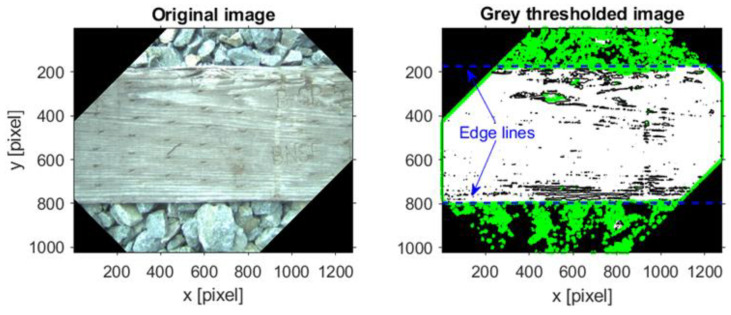
Estimation of the tie’s width in the pixel domain.

Images are in the pixel domain; therefore, a mapping factor should be established such that any measurement from the images is converted into the lengths in the common physical domain. The mapping factor was calculated through a calibration study. For this purpose, the widths of the first four ties were measured in the field. Next, the images corresponding to these ties were selected and the ties’ edges were marked on a polished grayscale version of the corresponding images (see Figure 11).

The distance between the edge lines was extracted from the images as the width of the ties in the pixel domain. The procedure was repeated three times for each tie to consider the uncertainty in processing the images and the average of the pixel domain widths was returned as the width of the ties. Equivalency between the in situ measurements and pixel domain estimations of the ties’ width revealed that each pixel is equal to 0.0130 inch.

**Figure 12:**
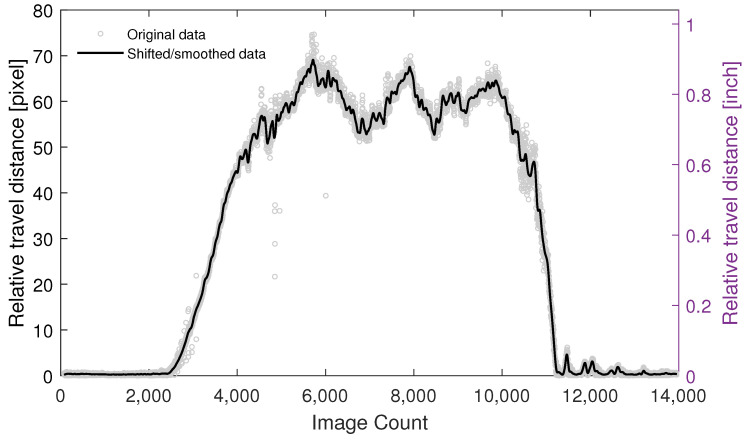
Relative travel distance for a test run. Each data point represents the relative travel distance of the corresponding image count with respect to the previous image count.

By tracking the travel distance between two successive images, the spatial resolution of the probing system is revealed, which returns the spatial position of the tracked ties. The acquisition system was programed such that each waveform is bundled to the corresponding image, returning a fully synchronized acquisition system. The trade-off between image resolution and frame rate was tuned such that a maximum acquisition frame rate of 100 frames per second (fps) was achieved. However, it was observed that some of the images were missing in the saving loop due to the buffer size limitations, imposed by the transmitting accessories and host computer. To maximize the ability of the image saving loop, a self-tuned queue-driven moderating variable was added to the developed LabVIEW program, which regulates the execution of the image saving loop such that the optimal number of missed frames are achieved without lowering the overall speed of the data acquisition system. Under such a configuration, the missed frames appeared in a one-in-between (i.e., alternate) scheme. To estimate the travel distance between two successive “saved” images, a relatively wide inspection region was selected in each frame and the speeded-up robust features (SURF) algorithm [41] was used to find blob features in each frame. The robust trackable points were automatically selected and tracked by Kanade–Lucas–Tomasi (KLT) tracker [42] through the next available frame. As a result of the relatively wide inspection region, many robust features were selected and tracked; however, some of them were recognized as invalid points in meeting the track reliability criteria. Such points were automatically detected and removed from the analyses. Moreover, an outlier analysis was performed on the reported pixel domain relative travel distances to reach a set of homogeneous data. It should be noted that dislocating the robust features because of local oscillation and sudden changes in the natural light or lens’s focus can add outliers to the data. Eventually, the average of the pixel-domain travel distances was computed as the travel distance between two successive saved frames. Repeating this procedure for all the existing frames, the relative displacement between successive saved images was achieved, as shown in Figure 12.

A local regression-based data-smoothing technique was also employed and a curve was fitted to the data. Moreover, a baseline shift was applied to the fitted curve to decrease the low-level oscillations imposed during initial waiting time (where the data acquisition system runs but the train is stationary).

As mentioned earlier, some of the captured images failed to be saved due to the buffer size limitations. Since there was considerable overlap between the successive saved images, the region of interest (ROI) of the missed frames can be retrieved using a “forward-backward” image inspection technique. Therefore, a complete tracking of the spatial position of the system is reconstructed, which is required to visualize the deflection profile of the scanned ties. Details of this technique are briefly summarized here. First, the ROIs of the saved images are isolated by placing a circle with a diameter equal to the effective head of a typical transducer. Since the missed frames appeared in alternate schemes, a linear interpolation could be a potential interpolator to apply to the extracted relative travel distances (in the pixel domain) to find the point representing the center of the missed ROI on the last saved image (i.e., the image right before the missed image). The accuracy of the retrieved ROI is examined through a “backward” inspection approach: On the first image saved next to the missed frame, a template ROI was gradually slipped back in a direction against the train movement to create the possible candidates of the missed ROI. Then, the structural similarity index (SSIM) [43] between the ROI retrieved by the forward inspection and each possible ROI was computed. If the SSIM returns a value greater than 90%, a complete similarity between the retrieved ROI and the candidate ROI is reported. A further calculation is also carried out to check if the estimated relative travel distance between the centers of the retrieved and the next available ROI matches with the corresponding portion of the travel distance adapted from the linear interpolation-based analysis. Employing the forward-backward inspection technique, all the missed ROIs were retrieved. The backward inspection revealed that the linear interpolation employed in the forward inspection is an acceptable assumption to retrieve the missed ROIs.

**Figure 13:**
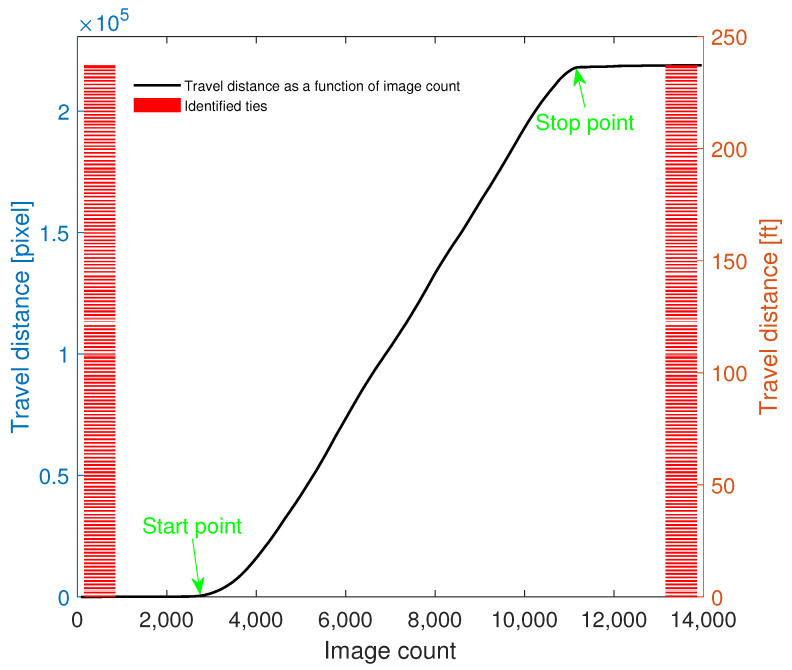
Image processing-based reconstructed travel distance and the obtained tie/ballast image classification results for a test run.

Images can also be used to develop a machine learning-based technique, aimed at classifying the probed objects. Such a technique can supplement the acoustic signal-based tie/ballast discrimination [44,45]. The classification is based on a texture analysis of the objects observed in the ROIs. Sample images of the ties and ballasts were randomly selected among all the images and a classifier was trained using the support vector machine (SVM) [46] method. In the present study, 75 images of the ties and 75 images of the ballasts were randomly selected among the captured images of the ties and ballasts during four independent runs at the BNSF yard to train and test an image classifier. In total, 106,560 features were extracted for each category (i.e., ties and ballasts) using the SURF algorithm. Then, the Bag-of-Words (BoW) [47] technique was employed to condense the features and reconstruct robust feature descriptors. The BoW is a technique widely used in natural language processing applications, such as topic modeling and text classification. The technique uses the k-mean clustering approach to iteratively group the descriptors into k clusters. Each cluster center represents a visual vocabulary, which is fed into the training core. In the present study, 80% of the strongest features for each category were selected to construct 500 visual vocabularies. Feeding the histogram of the visual vocabularies into the SVM, the image classifier was trained in a supervised manner. The confusion matrix of the test set returned an accuracy of 98.7%. In the next step, the image classifier was used to classify tie and ballast images and to demarcate the tie boundaries. A complete set of ROIs were recalled, and the classifier was used to analyze and classify the images. Figure 13 shows the obtained tie/ballast classification results (plotted on the image processing-based reconstructed travel distance) for one test run.

As mentioned in Section 4, there were 150 ties in the test tangent and all were correctly identified by the trained system. Note that in this figure, the travel distance has been plotted as a function of image count.

## 6. Results and Discussion

**Figure 14:**
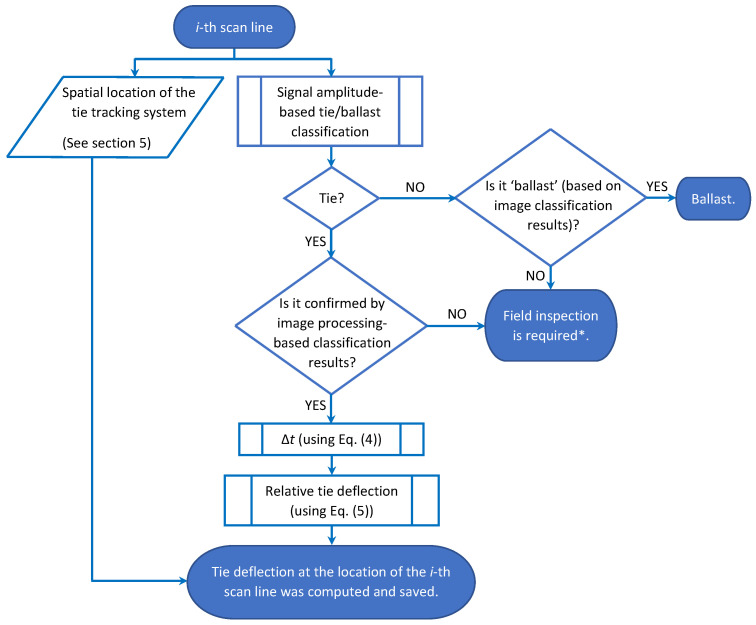
Flowchart of the proposed technique to track and compute the tie deflection at the location of the *i*-th scan line. ${}^{*}$ If the algorithm converges to this state, a field inspection is required to confirm the condition. Misalignment between the tie and the probing beam or misplacement of ballasts on top of a tie (in the camera’s ROI) can be some of the possible conditions.

Signal processing routines along with the image-processing techniques (outlined in Section 3 and Section 5) were used to compute the tie surface deflection profiles. Figure 14 shows the flowchart of the proposed technique to track and compute tie deflection along the *i*-th scan line.

**Figure 15:**
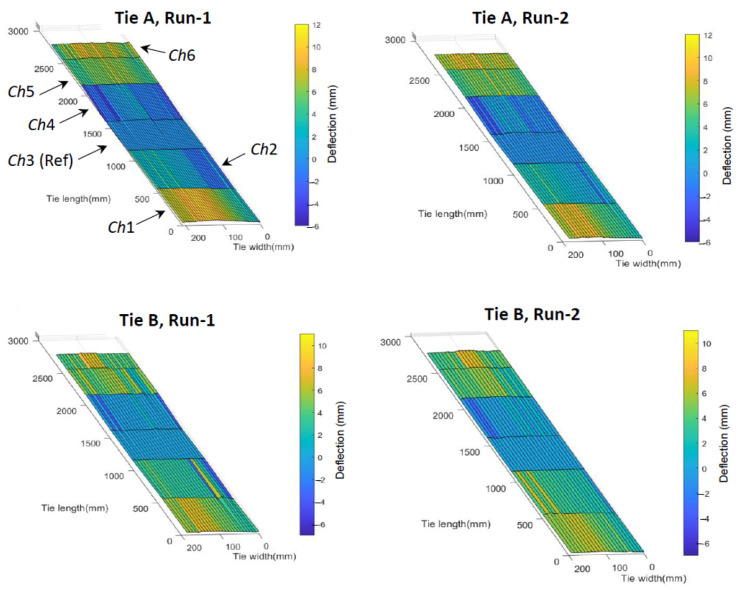
Full-field deflection profiles for two different ties in two independent test runs.

**Figure 16:**
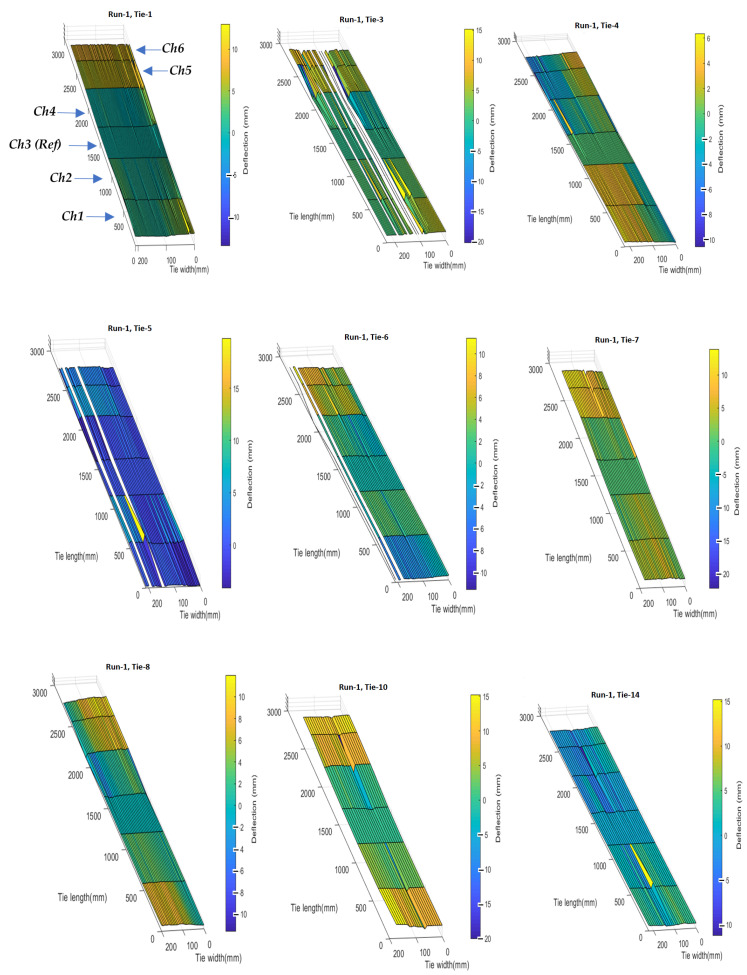
Full-field deflection profiles of nine ties scanned in a test run.

**Figure 17:**
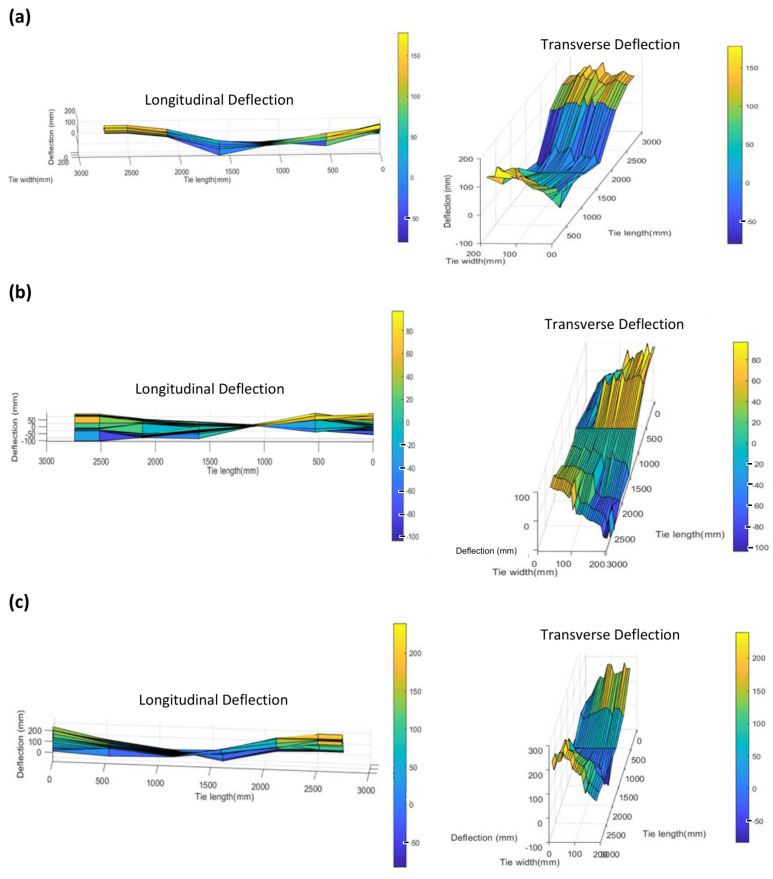
Longitudinal and transverse deflection profiles of three different ties with deflections scaled by 20×: (**a**) Tie I, (**b**) Tie II and (**c**) Tie III.

**Figure 18:**
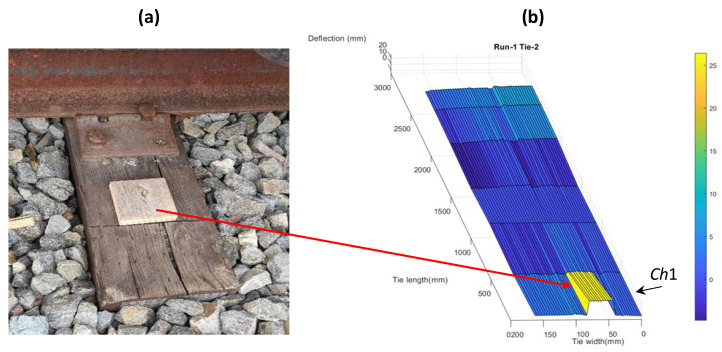
(**a**) Wood block placed on one of the ties and (**b**) 3D tie deflection profile with the wood block near transducer 1 (*Ch*1).

Figure 15, Figure 16 and Figure 17 and Figure 18b were created by depicting the tie deflection positions from the mid point (in mm and according to the indicated color scale) measured by each of the six air-couple transducers at each of the longitudinal scan lines along the car travel direction. Therefore, each tie is divided into six sections along the tie length (corresponding to the six transducers) and various scan lines along the tie’s width (corresponding to the various positions of the test car along the track). Figure 15 shows the 3D tie deflection profile for two ties (A and B) for two different independent test runs.

Figure 16 shows the 3D tie deflection profiles of nine ties scanned during one of the test runs.

The white regions in some of the ties indicate missed data points, where sufficient acoustic reflections were not achieved at all six transducers and therefore reliable measurements could not be obtained. Possible reasons for this could be the presence of the ballast on the tie surface or surface imperfections that result in wave scattering. A consistency between the reconstructed 3D profiles of the same tie for two different runs can be observed, indicating that the deflection measurements are reasonably precise. Figure 17 shows the longitudinal and transverse (twisting) deflections for three ties in one test run with the deflections scaled by 20× for better visualization.

From Figure 17, it is clear that both longitudinal deflections and tie cross-sectional twisting deformations can be obtained due to multiple measurements from different areas of the tie. It must be noted that walking test speeds allows a larger number of measurements along the tie width and fewer locations might be probed at higher testing speeds.

An analysis was performed to determine the accuracy of the measured deflections. A wooden block of known thickness was placed on one of the ties (control tie) in a way such that only transducer 1 (*Ch*1) could scan the plank. Figure 18a shows the block placed on the tie.

**Figure 19:**
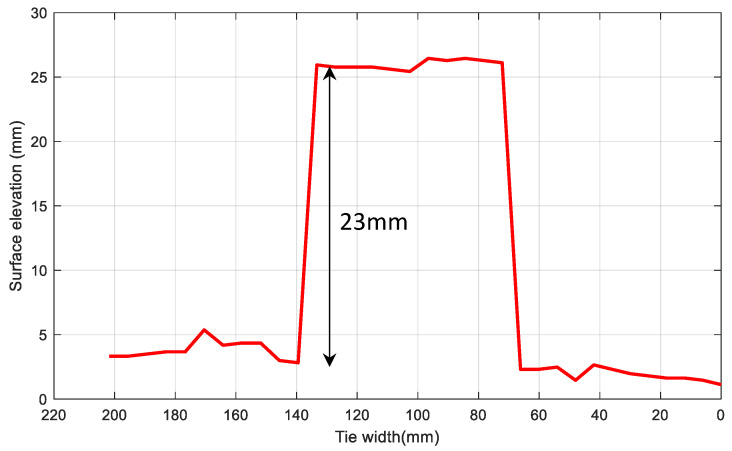
Tie width profile at transducer location 1 (*Ch*1) for a control tie with a wood block.

Figure 18b shows the 3D tie surface deflection for the control tie with the wood block. The wood block can be seen in the influence region of transducer 1 as an increase in surface elevation. Figure 19 shows the tie width profile at transducer 1 with the block appearing as a rise in the surface elevation over the width with respect to the baseline and again falling to baseline after transducer 1 completes scanning of the block.

The thickness of the wooden block could be computed from the deflection profile along the tie width at *Ch*1. A computed thickness of 23 mm was found to match the thickness of the block, showing that the displacement measurements are accurate. The apparent tapering of the wood block across the influence zone of transducer 1 (*Ch*1) in Figure 18b is an artefact produced by the linear interpolation performed between two transducer deflections to ensure continuity of the longitudinal deflection profile in the 3D reconstruction.

**Figure 20:**
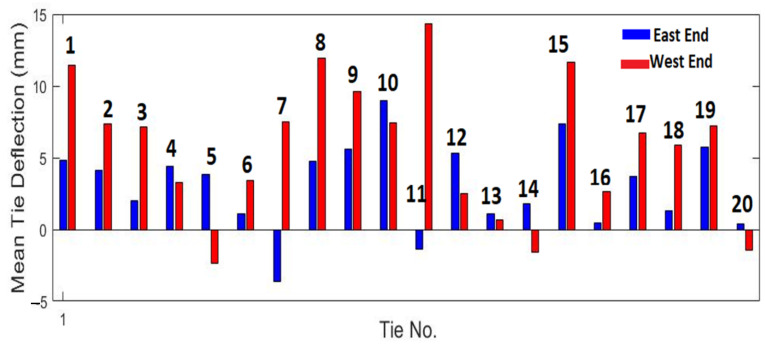
Mean tie end deflections of the first 20 ties computed over four test runs.

A statistical analysis was performed to evaluate the repeatability of the deflection measurements of the ties for different runs. The tie deflections at the two ends were tracked with respect to the central deflection for different ties and a mean and standard deviation of the deflections was computed over four different test runs. Figure 20 shows the mean tie deflections for the first 20 ties.

**Figure 21:**
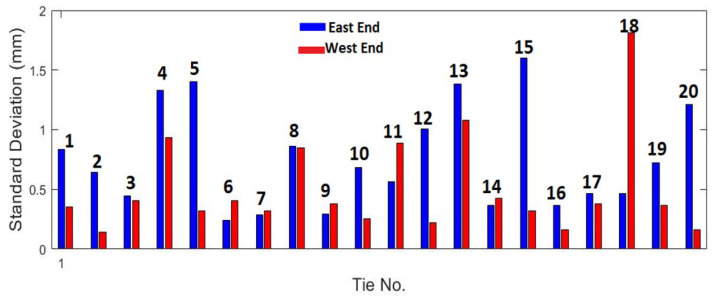
Standard deviation of tie end deflections of the first 20 ties computed over four test runs.

Figure 21 shows the standard deviation for the first 20 ties.

The standard deviations for most ties are less than 0.5 mm. Based on the repeatability tests, the deflection measurements at most ties are expected to lie within 1 mm (or two standard deviations) of the mean, indicating good precision when tests are repeated. Furthermore, note that when a tie has center-binding, the mean deflections at both ends of the tie (east and west) would be negative, or below the reference surface near the center. No ties with center-binding were found over the scanned section of the track in the tests.

## 7. Conclusions and Future Work

This paper introduced a technique and prototype under development for the measurement of full-field vertical and twisting deflections of railroad ties in motion. Once fully developed for higher speeds, the proposed technique could potentially allow structural monitoring of railroad ties and detection of center-bound ties during regular service runs at high speeds. Key signal processing routines, including time gating and cross-correlations, were introduced for accurately measuring the TOF of the airborne signals. Tests were conducted by mounting a prototype on a loaded train car at a BNSF rail yard. Preliminary results indicate the capability of measuring 3D tie deflection profiles at walking speeds. The testing parameters included validating the precision and accuracy of tie deflection measurements with the ultrasonic system at walking speed. Low testing speeds facilitated multiple deflection measurements along the tie width with high resolution. Higher testing speeds are expected to lower the resolution of twisting deformation measurements. For a representative of a 10 inch lift-off distance of the transducers from the top of the tie (satisfying clearance envelopes for non-contact testing) and a 9-inch standard tie width, the estimated total travel time of an airborne acoustic wave for probing one point of the tie is t= 1.48 ms. At a revenue speed of 60 mph, these calculations suggest that the same tie would be probed at six separate points along its width. Although this resolution would clearly be less favorable than the walking speed, it would theoretically still allow to extract 3D deflection information. Increasing the number of transducers in the sensing array would improve the resolution of longitudinal deflection measurements. The use of camera-acquired images to track spatial locations along the tie width was introduced as a convenient alternative to using an encoder, which requires contact with either the rails or wheels. An image-based analysis was also introduced as a method for demarcating the tie boundaries. The tests were conducted on a relatively new section of tangent track, where the old ties had reportedly been replaced with new ties about three years ago. Since most of the ties were new and pristine, the system was not able to flag any ties with center-binding support conditions during these tests. The results obtained during this preliminary study indicate that the precision and accuracy should be adequate for detecting ties with center-binding when train loads are sufficient to induce deflections. However, tests need to be conducted at higher speeds to determine if train-induced vibrations on the prototype mounting beam affect the measurements. One future avenue of research is as follows. Since the measurements of deflections are made only on the tie surface, pre-existing surface undulations may erroneously appear as tie deflections. Moreover, surface measurements alone may not fully indicate the extent of deformations on the bottom surface of the tie which is in contact with the ballast. To evaluate the “true deflections,” two transducer arrays (one placed near the axles that produce higher loads on the ties and one placed away from the axles producing lower loads on the ties) could be utilized. The difference in load factors at the the different locations of the arrays could be utilized to compute differential deflections to extract the “true deflections”. For example, the measurements from the array placed away from the axles could be used as a baseline and the measurements from the array located closer to the axle could be subtracted from the baseline measurements to obtain the “true deflections”. 

## Data Availability

The datasets generated during and/or analyzed during the current study are available from the corresponding author on reasonable request.
